# *Solanum betaceum* Fruits Waste: A Valuable Source of Bioactive Compounds to Be Used in Foods and Non-Foods Applications

**DOI:** 10.3390/foods11213363

**Published:** 2022-10-26

**Authors:** María Inés Isla, María Eugenia Orqueda, María Alejandra Moreno, Sebastián Torres, Iris Catiana Zampini

**Affiliations:** 1 Instituto de Bioprospección y Fisiología Vegetal (INBIOFIV-CONICET), San Miguel de Tucumán, Tucumán 4000, Argentina; 2 Biolates Network for Sustainable Use of Ibero-American Vegetable Biomass Resources in Cosmetics (BIOLATES, CYTED), Tucumán 4000, Argentina; 3 Facultad de Ciencias Naturales e Instituto Miguel Lillo, Universidad Nacional de Tucumán, San Miguel de Tucumán, Tucumán 4000, Argentina

**Keywords:** food waste, fruit waste, peel, seed, jelly, *Solanum betaceum*, phenolic enriched extracts, nutrients, phytochemicals, bioactive compounds

## Abstract

The fruit supply chain generates large amounts of waste that are often used as animal feed and in the production of both composts and fertilizers and biogas (anaerobic digestion). Since these types of procedures imply high economic costs related to drying, storage, and transport processes, more efficient and environmentally friendly utilization and recycling of this kind of waste are becoming significant for governments and industries. However, improper waste disposal increases the burden on the environment. Many of these fruit wastes, such as *Solanum betaceum* fruit waste, viz., peels, seeds, and pomace, could be considered potent bio-resource materials for several applications in the food and non-food industries due to their richness in valuable compounds. The basic composition of *Solanum betaceum* fruits seed has a high content of protein (20%), fiber (around 25%), sugar (11–20%) and low lipid content (0.4%), while *S. betaceum* peel has a low content of sugar (2–9%), protein (8–10%) and lipid (0.2–0.8%) and high fiber content (23%). Regarding the phytochemicals, the wastes have a high level of phenolics (0.2–0.6%) and pigments such as anthocyanins (0.06%). The inherent bioactive compounds of waste can be used as natural ingredients for foods, cosmetics, medicines, and the production of packaging materials production. Along this line, the present review covers all possible approaches for the valorization of *S.* *betaceum* waste in the food and non-food sectors.

## 1. Introduction

There is no global agreement on the definition of food waste. The United Nations’ Food and Agriculture Organization [1] makes a difference between food loss and waste, considering both as the decrease in the quantity or quality of food (only edible products) as a result of decisions made and actions taken between agricultural production and the arrival at retailers (for food loss), and between retail distribution and consumption (for food waste). However, throughout the food supply chain of fruits, significant amounts of inedible by-products considered wastes are generated and disposed of. In this context and in agreement with other authors, we shall be using a broader definition of food waste, including all edible and inedible parts of commodities and by-products that are discarded along every stage of the food supply chain between production and consumption [2,3,4]. Although the residues derived from the food supply chain represent a source of valuable resources, at the same time, they have a severe socioeconomic and environmental impact, especially in low-income and developing countries [3].

Food wastes can be of animal or vegetable origin, and within the latter, fruit waste can include seed, peel, rind, pomace, and pulp discarded during the whole food supply chain (about 25% to 30% of the commodity) [5]. According to the FAO, fruits are among the most consumed food worldwide, generating large amounts of waste and becoming an actual economic and environmental problem, yielding, together with vegetables, the highest losses and waste (edible products) among foods (about 60%) [5].

Fruit waste constitutes a valuable source of nutrients (sugar, proteins, vitamins, lipids, minerals) and bioactive compounds (polyphenols, carotenoids, dietary fibers, vitamins, and peptides, among others). Through various processes of valorization, these nutrients could be used as functional ingredients for food, cosmetics, drug manufacturing, and biomass; in this way, other valuable compounds such as enzymes, biopolymers, antibiotics, or biofuel could be obtained [3,6]. Among these fruits, many native fruits from South America, such as *Solanum betaceum* fruits and their by-products, can be highlighted. With a few exceptions, such as Colombia and Ecuador, they are underutilized in this region, but they constitute a reservoir of biofunctional components potentially profitable for the food, nutraceutical, pharmaceutical, and cosmetic industries.

**Figure 1:**
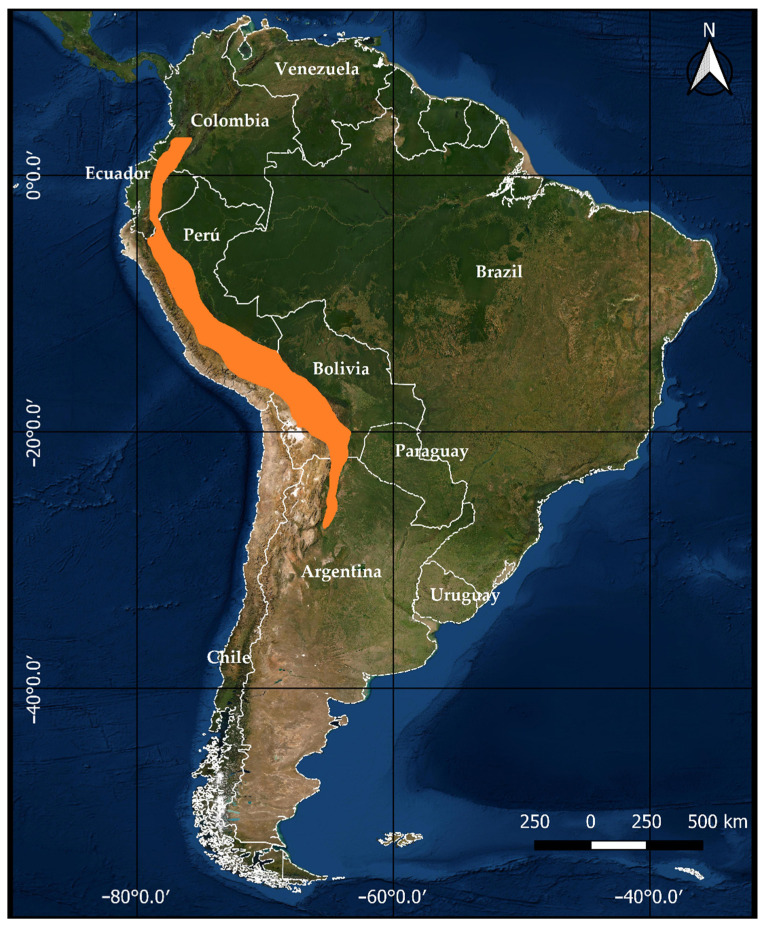
Geographic distribution of *S. betaceum* in South America. The orange section indicates the places of distribution. The map was made by the authors.

*Solanum betaceum* Cav. (named: tamarillo, chilto, or tree tomato) is a food species native to tropical or subtropical regions from Colombia to Argentina and belongs to the Solanaceae family. The wild cultivars exist in South American countries, including Argentina, Bolivia, Chile, Ecuador, and Peru (Figure 1). This species is classified as Vulnerable (VU). It is on the Red List of species at high risk of extinction in the wild, according to the IUCN (International Union for Conservation of Nature and Natural Resources) [7]. This fruit is cultivated in vast regions of South America mainly for local consumption, frequently in a processed form. Until 2009, Ecuador produced 25,000 tons in 3000 ha for domestic consumption at a rate of approximately 1.5 kg/capita/year [8]. In an extension of 6500 ha in Colombia, around 120,000 tons of *S. betaceum* are produced annually, most of which is consumed within the country [9]. According to the Ministry of Agriculture and Livestock of Ecuador [10], *S. betaceum* fruits rank tenth among inter-Andean fruit crops concerning crop yield and fifteenth regarding the extension of cultivated area. Regions exploited with this crop, i.e., cultivated and harvested areas, as the yield per hectare, have shown a growing trend in recent years. In the late 19th century, the fruit was globally introduced in Oceania (Australia and New Zealand), South-East Asia (India, Malaysia, Thailand, Indonesia, and Vietnam), Europe (Italy, Germany, Spain, Portugal, France, and the Netherlands) as well as in Africa (South Africa, Uganda, and Rwanda) [11]. New Zealand and Portugal are today the main producers and exporters. Meanwhile, Colombia started exporting fruit in 1990 (particularly to Europe), and currently, this is one of the five major fruits exported. North Island, New Zealand, produces 100 tons of *S. betaceum* fruits from about 100 ha, with a value of USD 2.4 million in domestic sales and USD 100,000 in export sales from New Zealand [12]. Countries like the United States, Japan, Hong Kong, Singapore, Australia, and the Pacific Islands are the prime international markets for *S. betaceum* fruits [13].

**Figure 2:**
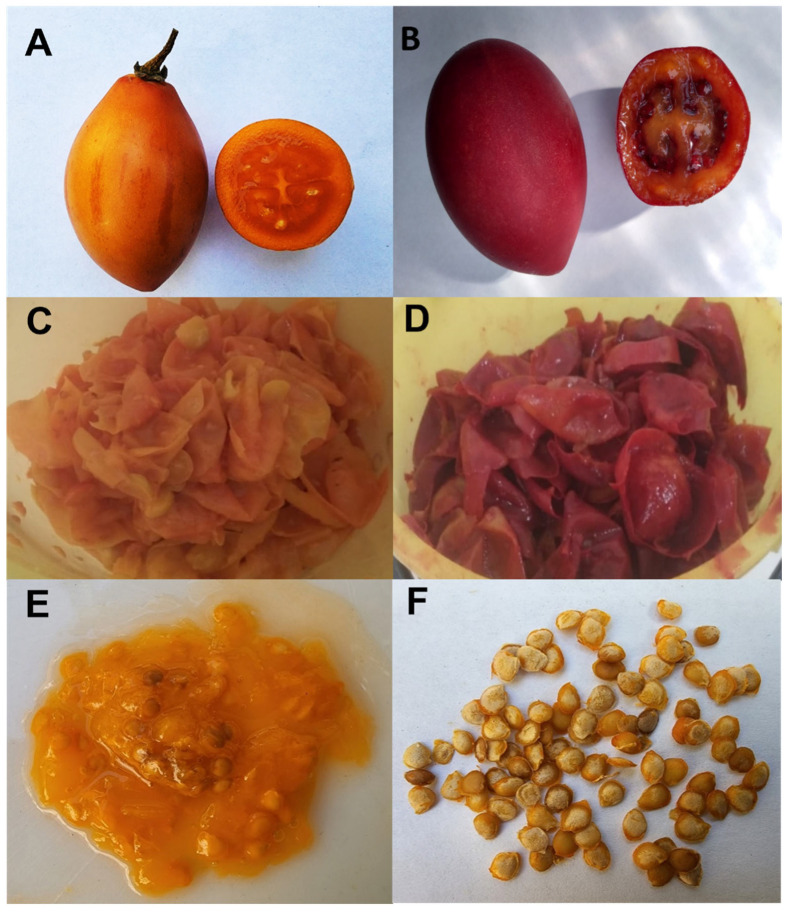
Pictures of (**A**) *Solanum betaceum* orange fruits; (**B**) *S. betaceum* red fruits; (**C**) peel of orange fruits; (**D**) peel of red fruits; (**E**) seed-jelly of orange fruits; (**F**) seeds of orange fruits. The pictures were taken by the authors.

*S. betaceum* fruit is a fleshy berry, juicy and bittersweet, with an ovoid to ellipsoid shape, and grows singly or in bunches. Different varieties are distinguished according to fruit color: red, orange, yellow and purple. The size of ripe fruits ranges between 4 and 10 cm in length, 3 and 6 cm in cross-section with a weight between 45 and 80 g (Figure 2). The pulp presents a firm texture with different colors, yellow, orange, red, and cream shades. It features many seeds (250) pubescent, covered with a gelatinous aril of different colors depending on the variety [14]. The *S. betaceum* fruits are used in food and medicinal preparations, as hypocholesterolemic and also applied topically against tonsil inflammation and for the treatment of anemia and liver and respiratory diseases [15].

The fruit of *S. betaceum* has been extensively studied in terms of its chemical composition, as well as its biological and functional activity. This fruit has numerous nutrients such as fiber (2.1–4.2%) and a high content of potassium (0.2–0.6%), ascorbic acid (0.01–0.04%) and carotenoids (0.2–5.2%), while components such as carbohydrates (0.1–1.9%) and lipids (0.01–0.7%) are present in smaller amounts (Table 1) [13,16,17,18,19,20,21,22]. Beneficial health activities of pulp have been reported as antioxidative, antiproliferative, antinociceptive, anti-inflammatory, allergenicity, anti-obesity, and antimicrobial properties [13,16,17,18,23,24]. Many of these reported properties are due to the presence of phenolic compounds (0.09–0.19%), such as derivatives of rosmarinic acid, 3-*O*-caffeoylquinic acid, or chlorogenic acid such as hydroxycinnamic acids identified in *S. betaceum* [16,17,18,23]. In the case of the red variety, the predominant polyphenolic compound in the pulp is anthocyanin, a hydrosoluble pigment (0.01–0.08%), principally delphinidin-3-rutinoside, while hexanoic acid methyl ester is the main volatile compound [24,25]. From *S. betaceum* pulp, numerous ingredients of interest in the food industry have been developed to prepare foods such as frozen functional pulps and energy drinks and also for the manufacture of ice creams [18,26,27]. Functional polysaccharides were extracted from Malaysian fruits and evaluated for their potential prebiotic and antinociceptive capacity [28]. The ripe *S. betaceum* fruits are consumed mainly in salads, jams, juices, and liquors [16]. During the process to obtain pulp and juices in Argentina, the seeds in the jelly, as well as the epicarp or peel or skin, are removed due to their bitter, sour, and astringent taste; the waste produced (peel, seed and jelly) can reach up to 49% of the processed fruit [16]. Not to mention that *S. betaceum* fruits of three cultivars from New Zealand [21] attest the peel-to-pulp weight ratio of approximately 1:6. These *S. betaceum* fruits by-products have increasingly been evaluated to obtain valuable compounds to be employed in the food, drink, packaging, bioadsorbent, cosmetic, pharmaceutical, and energy industries. However, the use of *S. betaceum* by-products is still limited; hence, more awareness and knowledge are needed to overcome traditional approaches for their disposal. The present review attempts to summarize fresh evidence on the food and non-food potential of *S. betaceum* waste, especially in the present circumstances, when the world is heading for a more environmentally friendly lifestyle.

Review process: This review was carried out by using a literature search on *Solanum betaceum*. Searches were conducted via the databases Science Direct (http://www.sciencedirect.com, accessed on 31 August 2022), PubMed (http://www.ncbi.nlm.nih.gov/pubmed, accessed on 30 August 2022), Google Scholar (http://scholar.google.com, accessed on 26 August 2022), Springer (https://link.springer.com, accessed on 29 August 2022), MDPI (https://www.mdpi.com, accessed on 31 August 2022), Scopus (http://www.scopus.com, accessed on 30 August 2022) and Scirus (http://www.scirus.com, accessed on 29 August 2022). Searches were also made using key words combinations of *Solanum betaceum*, *Cyphomandra betacea*, tamarillo, chilto, tree tomato, seed, peel (skin), jelly, biological activities, phytochemicals, anthocyanins, carotenoids, fatty acids, volatile compounds, hydrocolloids, food, cosmetic, food packaging, pharmaceutical, bioadsorbent, dye-sensitized solar cells, between others. The number of articles retrieved was 400. Sixty-five publications out of 400 have been used in the present review; the other 335 were not concerned with the potential uses of *S. betaceum* residues. Publications were considered up to the end of August 2022.

## 2. Nutritional and Phytochemical Composition of Powder Obtained from Waste of Red and Orange *S. betaceum* Fruits

The powder products obtained through grinding from wastes of *S. betaceum* fruits may be an alternative for their exploitation in food or non-food applications. Both the increase of particle surface area and the breakdown of the cell walls lead to improved bioavailability and bioactivity.

### 2.1. Nutritional Composition

The freeze-dried powders obtained from red and orange *S. betaceum* wastes (Figure 2) are a source of vitamin C (around 40 mg/100 g of peel and seed powder) and fiber (23.46 ± 2.00 to 29.5 ± 1.2 mg/100 g powder of peel and seed, respectively) (Table 2). Vitamin C content in New Zealand fruit peel was similar to the content reported in Argentinian *S. betaceum* peel and seeds (Table 2) [16,17,21].

Both seed and peel have low simple carbohydrate content. Sucrose is the dominant sugar in the peel and seeds of many cultivars [16,17]. The total sugar content in the peel of red *S. betaceum* from Argentina was similar to that reported in the peel of the red variety fruit from New Zealand, although the latter had a higher amount of fructose [20].

Pectins extracted from orange pulp of the *S. betaceum* variety were characterized in terms of their composition and functionality (highly methoxylated homogalacturonans mixed with type I arabinogalactans) [29,30,31]. Recently, a hydrocolloid was also obtained from red *S. betaceum* peel and seed jelly, a waste product of *S. betaceum* (Figure 2). The polysaccharide was more branched and less viscous [32] compared to the pulp hydrocolloid and is highly related to polar pigments (anthocyanins) [30].

Regarding the protein content, it was found that fruit seeds from Argentina contained around 20% [16,17].

Otherwise, *S. betaceum* peel extractions presented the oleic acid as the predominant fatty acid, whereas in the seed extract, the linoleic acid was the predominant (Table 3) [33,34,35]. Oleic (14.93%), palmitic (9.41%), stearic (2.23%), and linolenic acids (1.73%) were also reported (Table 3). The percentage of total polyunsaturated fatty acids in this seed oil was 72.20%, which makes this seed oil an interesting source of essential fatty acids for both food and cosmetic and pharmaceutical applications. *S. betaceum* seed oil is much richer in essential fatty acids than commercial seed oil [34].

### 2.2. Phytochemical Composition

The content of total phenolic compounds (TPC) in Argentinian red *S. betaceum* peel and seeds powder was 408.9 ± 2.3 and 623.6 ± 1.6 mg GAE/100 g DW, respectively, while in orange fruit peel and seeds was 523.8 3 and 179.4 3 mg GAE/100 g DW, respectively (Table 2) [16,17]. Similar results were reported for the peel of the purple variety of *S. betaceum* from Ecuador and Malaysia, while the peel of fruits from New Zealand presented a higher amount of TPC (Table 2) [19,36,37]. The cultivar type and geographical distribution also significantly affect the percentage of phenolics in waste.

Two main compounds, rosmarinic and caffeoylquinic acids, were identified in both genotypes from Argentina Yungas and in purple and yellow varieties from Ecuador [16,17,23], but in orange *S. betaceum* fruits, the diversity of phenolic compounds in pulp, peel and seeds were greater (12 caffeic acid derivatives and related phenolics, 10 rosmarinic acid derivatives and 7 flavonoids) [16]. The total content of each phenolic acid (rosmarinic and caffeoylquinic acids) in red *S. betaceum* peel powder was 0.291 ± 0.001 g/100 g powder and 0.354 ± 0.002 g/100 g powder, respectively. The content of flavones and flavonols was higher in the peel of the *S. betaceum* orange variety from Argentina compared to the red variety. The flavonoid content in seed was similar in both varieties. On the other hand, the fruit peel from Malaysia was similar in the content of flavonoids to Argentinian fruits (Table 2) [16,17,36]. The red *S. betaceum* waste powders contain condensed tannins, mainly seed powder (265.0 mg/100 g DW, Table 2).

Anthocyanins are other phenolic compounds present in red *S. betaceum* waste powder [16,17,36,37,38]. The pigment content was 62.5 and 65.2 mg EC/100 g DW for the peel and seed, respectively, in red *S. betaceum* fruits from Argentina but non-detected on orange *S. betaceum* peel and seed [16,17]. The content of these pigments was approximately 2.5 to 4.1 times higher in the peel of *S. betaceum* red and purple fruits from New Zealand, while the peel and seed jelly of fruits from Colombia turned out to be lower than those from Argentina (Table 2) [36,37,38]. Another report indicates that the anthocyanins of the Colombian *S. betaceum* peel were more stable to changes in pH than those of the seed-jelly; this could be due to a large number of polymeric anthocyanins, as well the possible presence of several color stabilizer compounds, such as organic acids, phenolic acids, flavonols, and flavonols in *S. betaceum* fruits peel [38].

Inversely, carotenoids were only detected in the seeds and peels of the *S. betaceum* orange variety from Argentina but not in red fruits (Table 2) [16,17]. Several carotenoids were identified in the peel of Brazilian fruit, with β-carotene being the majority [39]. A more detailed characterization of these pigments in the fruit of *S. betaceum* from Brazil was carried out by using HPLC-PDA-MS/MS techniques after saponification [40]. Xanthophylls esterified with palmitic and myristic acids were identified for yellow *S. betaceum* from Ecuador. All-trans-β-carotene and all-trans-β-cryptoxanthin esters were the main carotenoids in this variety [41]. The content of carotenoids in the orange variety of fruits from Argentina was similar to that reported for the peel of fruits from Malaysia and New Zealand (Table 2) [16,17,21,36].

## 3. Functionally Active Ingredients from *S. betaceum* Wastes to Food and Cosmetic Products

### 3.1. Polyphenol-Enriched Extracts from Seed, Peel and Pomace of S. betaceum Fruits to Elaborate Food Formulations with Biological Activity

**Figure 3:**
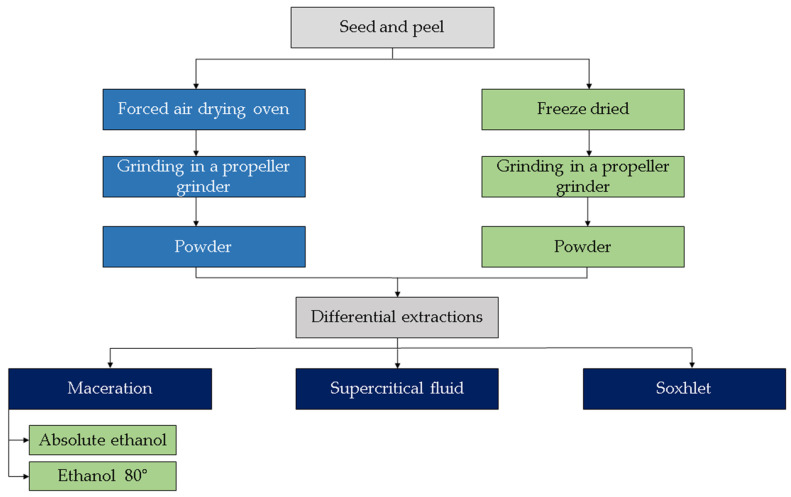
Scheme of the extraction procedure of phytochemicals from the seed and peel.

Polyphenol-enriched extracts (PEE) were obtained from grounded and dried seeds and peels of red and orange *S. betaceum* fruits grown in Argentine Yungas. The product was elaborated using ethanol 80° as solvent by maceration assisted by ultrasound (Figure 3). The HPLC-MS/MS analysis of PEE allowed the identification of 11 caffeic acid derivatives and related phenolic compounds, 8 rosmarinic acid derivatives, and 5 flavonoids (Table 4) [16,17]. The PEE has numerous biological properties such as hypoglycemic activity, inhibiting the activity of enzymes responsible for the breakdown of carbohydrates and lipids (α-glucosidase, α-amylase, and lipase), anti-inflammatory activity and antioxidant activity by various mechanisms [16,17,27,42]. Polyphenols extracted from *S. betaceum* wastes (peel and seed) could counteract subclinical oxidative stress in the gastroduodenal tract by scavenging free radicals, thus reducing intestinal inflammation induced by a high-fat diet. Polyphenols could also modulate the bioavailability of ingested nutrients and body weight through the activity of digestive enzymes [16,17]. This by-product is a promissory resource that could be used as a food, a functional food ingredient or a nutraceutical. Other authors reported ferulic acid and sinapic acid in peel and chlorogenic acids in *S. betaceum* peel and seed [33]. These differences could be related to the different solvents used for phenolic extraction. Ethanol 80°, absolute ethanol, and acetone were used as extraction solvents [16,17,33]. The extract in absolute ethanol obtained from fruits purchased at the local market in Bogotá (Colombia) also showed antioxidant activity and hypoglycaemic potential [33].

Extracts from *S. betaceum* fruit peel from Alimentos SAS (Bogotá, Colombia) were also obtained by supercritical fluid extraction (SFE) and Soxhlet extraction (SE), but their chemical composition has not been described hitherto (Figure 3). The highest antioxidant activity in a trial when using cooked beef was demonstrated by the extract obtained by SFE with CO_2_/EtOH (50 °C/30 MPa and 2% EtOH) [43]. Sanchez et al. [44] reported antioxidant activity for peel and seed extracts obtained through different extractive procedures. Other authors obtained aqueous extracts from the peeled fruits of *S. betaceum*, where the main components were compounds derived from hydroxycinnamic and hydroxybenzoic acids, flavonol glycosides, and anthocyanins (rutinosides of delphinidin, cyanidin, pelargonidin). These extracts were encapsulated in cubosome nanoparticles and incorporated into yogurt with the consequent improvement in physicochemical and nutritional properties, as well as antioxidant activity [45].

### 3.2. Polyphenol-Enriched Extracts from Seed, Peel, and Pomace of S. betaceum Fruits to Obtain Cosmetic Formulations with Biological Activity

The hydrolytic enzymes, i.e., tyrosinase, elastase, collagenase, and hyaluronidase, are overexpressed during the aging process in the skin. These hydrolytic enzymes are involved in the regulation of skin hyperpigmentation, dryness, wrinkles, or elasticity. The PEE of *S. betaceum* peel and seeds inhibited the four enzymatic activities, particularly elastase and tyrosinase. The results suggested that *S. betaceum* fruit sub-products can be considered a source of bioactive phenols with a promising future in the cosmetic industry for uses such as hydrogels, lotions or creams [46]. These products represent a feasible (sustainable, not expensive, but efficient) alternative to plant-derived extracts, which are more commonly used in cosmetic formulations.

### 3.3. Bioactive Carotenoid-Enriched Extracts from Seed and Peel of S. betaceum Fruits

Carotenoids were extracted from *S. betaceum* fruit peel by using ethanol, dried at 60 °C and powdered in a blade mill. The antioxidant extract obtained was microencapsulated by spray-drying polymers [47]. Microcapsules of carotenoids from *S. betaceum* pulp were also obtained by spray-drying [48]. When Mertz et al. [49] studied the changes in carotenoid contents after the thermal pasteurization of *S. betaceum* nectars, two conclusions were reported; namely, the zeaxanthin esters were the least thermolabile carotenoids, and the level of dissolved oxygen did not have an influence on their degradation.

### 3.4. Protein from Solanum betaceum Fruits

A thorough analysis of antioxidant activities against free radicals and the genotoxic/antigenotoxic effects of proteins was carried out. These proteins, with a molecular mass of around 17 kDa purified from *S. betaceum* fruits (cyphomine), were xanthine oxidase enzyme inhibitors; they prevented the formation of uric acid and reduced oxidative damage by scavenging hydroxyl radicals and superoxide anions in a dose-dependent manner. After heat treatment at 80 °C, the antioxidant activity of cyphomine was preserved, and no genotoxic and mutagenic effects were observed [50,51]. The benefit of these proteins as ingredients for the development of functional foods will be crucial as regards the impact on human health, not to mention their importance as a source for the production of bioactive peptides.

### 3.5. Oil from S. betaceum Seeds to Be Used in Food or in Cosmetics

The oil from *S. betaceum* seeds was rich in fatty acids such as linoleic and oleic acids [34]. Although it had a high antioxidant capacity and low acidity, a high peroxide value was reported [52,53]. The prevention of different disorders, particularly cancer, heart disease, and cardiovascular disease, as well as a treatment for hypertension, diabetes, and inflammatory, autoimmune and thrombotic diseases, have been reported as some of the health benefits of polyunsaturated fatty acids [54]. Besides, linolenic acid can provide all the assets of the sought-after essential omega-3-rich oil in food or cosmetic products, namely moisturizing cream or gels [55], or else, as an alternative source of omega-3 supplements without the somewhat disagreeable fish aroma. Thus, this new source of essential fatty acids proves to be of good value for both the food industry and cosmetic and pharmaceutical applications.

### 3.6. Volatile Compounds (VC) in Peel of Freeze-Dried S. betaceum Fruits

Fruit volatiles are the result of various chemical groups, like alcohols, aldehydes, esters, ketones, furanones, and terpenes, formed from precursors as the fruit ripens and changes postharvest [56]. Out of 121 features, peel had two fewer volatile compounds than pulp (115 compounds compared to 117). These VC were further classified into their chemical groups based on structure: ketones, esters, fatty acids, nitrogen compounds, furans, aldehydes, alcohol, benzenes, hydrocarbons, carboxylic acids and derivatives, sulfur compounds, terpenes, and a pyran compound. The main volatile compounds present in the peel were 4-hydroxy-4-methyl-2-pentanone; 3,5-dihydroxy-2-methyl-4H-pyran-4-one, and phthalic acid, hept-4-yl isobutyl ester, whereas 5-hydroxymethylfurfural, 3-furaldehyde, and 3,5-dihydroxy-2-methyl-4H-pyran-4-one were the major abundant VC for the pulp tissue. The major contributor to the overall flavor of *S. betaceum* was methional, frequently associated with tomato-like flavor notes, and perhaps this flavor may account for the original name of the *S. betaceum* fruits. Maltol is known to enhance the oral bioavailability of gallium [57], as are iron-based drugs [58], which adds to the potential of use as a therapeutic adjunct. A higher concentration of maltol and its derivatives can be found in the pulp rather than in the peel of *S. betaceum* [24]. The flavor of foods as flavoring agents or fruit flavor additives can be greatly improved by using methional and maltol. As highlighted, the peel, which is often discarded as a by-product, has great potential to be used as a natural preservative to enhance the flavor and shelf life of other food products in freeze-dried form.

### 3.7. Hydrocolloid from S. betaceum Pulp and Seed Mucilage

Several studies have reported on the health benefits and technological versatility of fruit-derived hydrocolloids. These benefits include water-holding, oil-holding and foaming capacity, foam stability, and emulsifying activity that could be used in the food, pharmaceutical, or cosmetic industry. Gannasin et al. [59] extracted and characterized a hydrocolloid from *S. betaceum* puree, i.e., the combination of pulp and seed mucilage. Hydrocolloids show good oil-holding capacity (2.11 g oil/g dry sample), emulsifying activity (84.74%), and emulsion stability (94%), whereby it could be applied as a plant-based emulsifier for both the food and cosmetic industries. Emulsifiers allow for achieving the desired viscosity, as well as a texture that is beautiful and pleasant on the skin in cosmetics. In addition, hydrocolloids allow the creation of structural systems that will give meaning to the final application of the cosmetic, as is the case with masks or bath gels. Besides that, it demonstrated a foaming capacity of 32.19%, which is unique for a plant-derived hydrocolloid. These hydrocolloids showed better foam stabilization characteristics (79.36% of initial foam volume) than bovine gelatin (11.01%) at 2 h from foam formation. These findings suggest that these polysaccharides can be used as functional ingredients in foods such as low methoxyl pectin in reduced sugar products, milk gels and desserts [59,60].

## 4. Innovations in Biodegradable Food Packaging Manufacture by Using Polyphenols, Pigments and Polysaccharides from *S. betaceum* Fruit Waste

Petroleum-based polymeric packaging is generally not degradable, and only a portion is recycled. Discarded plastics pollute the environment causing severe damage to the health of living beings; in many cases, even microplastics have been found in the organs of both humans and animals. In this sense, sustainable packaging through the use of biodegradable polymers and natural ingredients, such as plant-based food waste and by-products, is an excellent alternative to reduce waste accumulation leading to a positive impact on the environment. Electrospinning technology has greatly improved polymer processing with its ability to convert solutions of bioactive compounds and charged biopolymers into packaging structures. Thus, zein fibers containing extracts enriched with polyphenols from the seeds and peel of *S. betaceum* fruits have been obtained. These fibers were deposited on polyhydroxyalkanoate (PHA) films using the electrospinning technique. Crosslinking allowed a gradual release of phenolic compounds in foods (rosmarinic and caffeic acids and their derivatives), maintaining their antioxidant properties [61]. Recently, films containing anthocyanin-enriched extracts obtained from the peel of *S. betaceum* red fruits were obtained by casting [32]. The pectin-enriched extract obtained from the peel of *S. betaceum* was used as a film-forming matrix, and the bioactive extracts were incorporated. To verify its antioxidant activity, salmon fillets were coated with the developed films, showing that they extended shelf life by reducing the oxidation of biomolecules for more than a week of storage under refrigeration conditions [32]. These composite films were made directly from by-products obtained from *S. betaceum* waste.

## 5. Bioremediation: Bioadsorption of Toxic Metals by *S. betaceum* Peel Waste

Bioadsorption is a process that allows the uptake of metal ions due to the property of some biomasses (alive or dead) of binding and accumulating this type of pollutants through different mechanisms. [62]. The use of metallic components in the industry has resulted in an increase in the residues produced, especially liquid ones. Not treated correctly, these residues cause various serious problems, such as the contamination of rivers, lagoons, and seas, affecting the entire ecosystem. In this framework, the bioadsorption capacity of by-products such as *S. betaceum* peel on plumbum and chromium ions was evaluated [63]. The author concluded that the *S. betaceum* peel presented good adsorption values for both ions, although with the highest affinity for lead. In addition, the bioadsorbents can be reused after the heavy metal removal process.

## 6. Dye-Sensitized Solar Cells by *S. betaceum* Pulp and Seeds

Dye-sensitized solar cells (DSSC) are a promising and economical alternative to converting solar energy into electrical energy. Currently, there is a tendency to use natural dyes for these cell manufacturing, which have the advantage of low production cost, flexibility, easy preparation and biodegradability [64]. Susanti et al. [65] developed DSSC using red tamarillo pulp extracts coupled with TiO_2_. The DSSC presented an efficiency of 0.043% due to the high active surface. However, despite the low efficiency, the work demonstrated the ability of *S. betaceum* extracts to develop promising low-cost DSSC.

## 7. Conclusions

**Figure 4:**
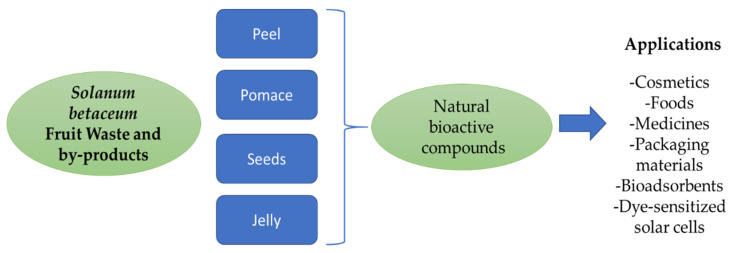
Applications of *Solanum betaceum* fruits waste.

The fruit supply chain generates large amounts of by-products that are underutilized, and their accumulation has a high socioeconomic cost and an impact on the environment. These wastes may have a variety of compounds with biofunctional value with proven health-beneficial effects, such as anti-inflammatory, antioxidant, anti-cancer, and anti-obesity phytochemicals, or biotechnological applications, such as antioxidant and antimicrobial phytochemicals, colorants and pigments, volatile aroma compounds, pectins, or biopolymers, among others. With this antecedent, the present work review has explored in depth the opportunities *S. betaceum* fruit by-products offer as a sustainable source of bioactive substances to be employed in functional food, pharmaceutical, and cosmetic products, innovative packaging, and bioremediation applications. Peels, seeds, pomace, and jellies from *S. betaceum* fruits are a source of innumerable bioactive substances, even in amounts that can exceed their content in the pulp (Figure 4).

Despite New Zealand, Portugal, Colombia, and Ecuador being the leading producers of tree tomato fruits for local consumption or exportation to countries like the United States, Japan, Hong Kong, Singapore, and Australia, the wastes of this fruit are not a global problem. However, the valorization of by-products of native fruits such as *S. betaceum,* the trade of which is limited in many regions, may offer the chance to promote the commercial exploitation of these natural resources in a circular economy model through their sustainable integral use. Nevertheless, to guarantee the exploitation of the whole *S. betaceum* fruit, research efforts should be focused on overcoming economic and technological drawbacks related to the extraction and purification of the natural ingredients present in this fruit. Low-cost, less energy-demanding, and high-efficient separation processes that ensure a maximum yield of bioactive compounds at an industrial scale are critical for achieving the integral use of *S. betaceum* fruits. In this regard, new and non-conventional environmentally friendly extraction technologies should also be explored. Additionally, studies on the economic feasibility of *S. betaceum* fruit waste utilization should be performed to guarantee the sustainable exploitation of this native fruit.

## Figures and Tables

**Table 1:** Phytochemical and nutritional composition of different varieties of *S. betaceum* fruits.

Nutritional and Phytochemical Composition	Orange, Yellow and Golden Yellow Fruits			Red and Purple Red Fruits		
	Orange Fruits (Argentina) [16]	New Zealand (Yellow) [13]	Ecuador (Golden-Yellow) [19]	Argentina (Red) [17]	New Zealand (Red) [13]	Ecuador (Purple-Red) [19]
Total phenolic (mg GAE/100 g FW)	89.1	117.0	125.0	68.1	191.0	187.0
Flavone and flavonol (mg QE/100 g FW)	20.4	-	-	25.1	-	-
Anthocyanins (mg C-3GE/100 g FW)	0.2	ND	ND	12.7	82.0	38.0
Ascorbic acid (mg AA/100 g FW)	15.2	31.0	17.0	15.4	29.8	16.0
Carotenoids (g β carotene/100 g FW)	0.2	-	3.4	0.2	-	5.2
Total sugar (g GE/100 g FW)	1.1	3.4	-	2.1	3.5	-
Glucose (g/100 g FW)	0.1	0.8	1.7	0.2	0.8	1.4
Fructose (g/100 g FW)	0.3	0.9	1.6	0.3	0.9	1.4
Sucrose (g/100 g FW)	1.0	1.6	1.9	1.5	1.7	1.7
Total Protein (g/100 g FW)	2.4	1.9	2.4	2.2	2.0	2.2
Fat (g/100 g FW)	0.02	0.5	0.7	0.04	0.4	0.6
Fiber (g/100 g FW)	2.1	3.2	-	4.2	3.3	-
Potassium (mg/100 g FW)	637.8	292.0	398.0	251.8	321.0	379.0
Iron (mg/100 g FW)	0.2	0.4	0.2	-	0.5	0.4
Magnesium (mg/100 g FW)	21.54	20.0	16.0	15.5	21.0	14.0

ND: Not detected.

**Table 2:** Phytochemical composition of *S. betaceum* wastes.

Phytochemical Content of *S. betaceum* Wastes	Red and Orange Fruit Peel (Argentina)	Red and Orange Fruit Seeds (Argentina)	Purple and Golden Yellow Peel (Ecuador)	Purple and Golden Yellow Seed-Jelly (Ecuador)	Red Fruit Peel (Colombia)	Red Fruit Jelly (Colombia)	Fruit Peel (Malaysia)	Fruit Seeds (Colombia)	Yellow, Red and Purple Fruits Peel (New Zealand)
Total phenolics (mg GAE/100 g)	408.9 [17] 523.8 [16]	623.6 [17] 179 [16]	620 [19] ^a^ 387 [19] ^a^	152 [19] ^a^ 94 [19] ^a^			240–489 [36]		1583.8 [37] 1673.28 [37] 2225.1 [37]
Flavone and flavonols (mg QE/100 g)	195.3 [17] 265.7 [16]	180.5 [17] 175.6 [16]					129–336 [36]		
Condensed tannins (mg procyanidin B2/100 g)	75.7 [17] ND [16]	265.0 [17] ND [16]							
Anthocyanins (mg C-3GE/100 g)	62.5 [17] 1.01 [16]	65.2 [17] ND [16]			0.20 ^1^ [38]	20.03 ^1^ [38]	0.61–1.36 [36]		1.24 [37] 155.82 [37] 259.18 [37]
Ascorbic acid (mg AA/100 g)	43.5 [17] 51.1 [16]	45.2 [17] 56.8 [16]							22 [21] ^a^ 18 [21] ^a^ 19 [21] ^a^
Carotenoids (g β carotene/100 g)	ND [17] 1.37 [16]	ND [17] 0.53 [16]					0.01–0.02 [36]		0.23 [21] ^a^ 0.15 [21] ^a^ 0.15 [21] ^a^
Total sugar (g GE/100 g)	8.9 [17] 2.23 [16]	20.5 [17] 11.9 [16]							3.6 [20] 9.7 [20] 6.9 [20]
Glucose (g/100 g)	0.6 [17] 0.9 [16]	0.9 [17] 0.7 [16]							1.3 [20] 3.6 [20] 2.5 [20]
Fructose (g/100 g)	1.2 [17] 2.7 [16]	3.9 [17] 1.9 [16]							1.8 [20] 5.1 [20] 3.6 [20]
Sucrose (g/100 g)	4.2 [17] 7.8 [16]	8.5 [17] 6.8 [16]							
Total Protein (g/100 g)	10.5 [17] 8.8 [16]	21.9 [17] 20.9 [16]							
Fat (g/100 g)	0.8 [17] 0.2 [16]	0.4 [17] 0.3 [16]						17.4 ^2^ [35]	
Fiber (g/100 g)	23.2 [17] 23.4 [16]	29.5 [17] 28.42 [16]							

The superscript “^a^” indicates that the results are expressed in fresh weight (FW), and the rest of the values in the table are expressed in dry weight (DW); ^1^: mg delphinidin 3-glucoside/L; ND: Not detected; ^2^: Colombian tamarillo seed oil.

**Table 3:** Fatty acids composition of *S. betaceum* seed oil.

Type of Fatty Acid	% of Fatty Acid
Oleic (C18:1 n-9)	17.20 ± 0.60 [33] 14.93 ± 0.44 [34] 15.11–17.94 [35]
Linoleic (C18:2)	58.30 ± 1.00 [33] 70.47 ± 0.35 [34] 66.67–72.09 [35]
Linolenic (C18:3)	1.73 ± 0.04 [34] 1.53–3.50 [35]
Palmitic (C16:0)	9.41 ± 0.15 [34] 7.70–11.02 [35]
Palmitoleic (C16:1)	0.54 ± 0.05 [34] 0.19–0.25 [35]
Stearic (C18)	2.23 ± 0.12 [34] 1.42–3.50 [35]
Arachidic (C20:0)	0.23 ± 0.06 [34]
Phellonic (C22:0)	0.22 ± 0.01 [34]
Lignoceric (C24:0)	0.23 ± 0.14 [34]

**Table 4:** Phenolic compounds identified in seeds and peel of *S. betaceum* fruits by HPLC MS/MS.

*S. betaceum* by-products	Identification of Phenolic Compounds
Seeds and peel polyphenol enriched extract	Caffeoyl hexoside
	Dicaffeoylquinic acid derivative
	Caffeoyl quinic acid
	Caffeoyl quinic acid hexoside
	Dihydrokaempferol pentoside
	Caffeoylquinic acid
	3-Caffeoyl quinic acid
	Apigenin pentoside
	Hydroxy rosmarinic acid
	Synapoyl hexoside
	Rosmarinic acid dihexoside
	Quercetin rhamnoside
	Rutin
	Rosmarinic acid hexoside
	Isorinic acid hexoside
	Malonyl rosmarinic acid hexoside
	Quercetin hexoside
	Kaempferol rutinoside
	Quercetin glucuronide
	Rosmarinic acid
	Isorinic acid

## Data Availability

The datasets generated during and/or analyzed during the current study are available from the corresponding author upon reasonable request.

## Abbreviations

PEE: Phenolic enriched extracts, HPLC-DAD: HPLC coupled to a diode array detector, HPLC-PDA-MS/MS: HPLC coupled to a diode array detector and mass, SC50: 50% scavenging capacity, GAE: Gallic acid equivalent, QE: Quercetin equivalent, C-3GE: Cyanidin-3 glucoside equivalent, GE: Glucose equivalent, DW: Dry weight, FW: Fresh weight.
